# Citizens’ Adherence to COVID-19 Mitigation Recommendations by the Government: A 3-Country Comparative Evaluation Using Web-Based Cross-Sectional Survey Data

**DOI:** 10.2196/20634

**Published:** 2020-08-11

**Authors:** Abrar Al-Hasan, Dobin Yim, Jiban Khuntia

**Affiliations:** 1 College of Business Administration Kuwait University AlShadadiya University City Kuwait; 2 Loyola University Maryland Baltimore, MD United States; 3 University of Colorado Denver Denver, CO United States

**Keywords:** COVID-19, adherence, social distancing, government perception, information sources, social media, knowledge

## Abstract

**Background:**

Social distancing is an effective preventative policy for the coronavirus disease (COVID-19) that is enforced by governments worldwide. However, significant variations are observed in following the policy across individuals and countries. Arguably, differences in citizens’ adherence actions will be influenced by their perceptions about government’s plans and the information available to guide their behaviors—more so in the digital age in the realm of mass influence of social media on citizens. Insights into the underlying factors and dynamics involved with citizens’ adherence process will inform the policy makers to follow appropriate communication and messaging approaches to influence citizens’ willingness to adhere to the recommendations.

**Objective:**

The aim of this study is a comparative evaluation of citizens’ adherence process to COVID-19–relevant recommendations by the government. The focus is on how three different countries’ (United States, Kuwait, and South Korea) citizens, randomly sampled, respond to governments’ pandemic guidance efforts. We draw insights into two categories of perceived government roles in managing the pandemic: (1) citizens’ perceptions of government’s role in responding to the pandemic and (2) citizens’ perceptions of government’s business reopening efforts. Undoubtedly, the internet and social media have burgeoned, with differing effects on shaping individuals’ views and assessments of the COVID-19 situation; we argue and test for the effects of information sources, social media use, and knowledge on the adherence actions.

**Methods:**

We randomly sampled web-based survey data collected by a global firm in May 2020 from citizens of the United States, Kuwait, and South Korea. A nonlinear ordered probit regression, controlling for several counterfactuals, was used for analysis. The focal estimated effects of the study were compared across countries using the weighted distance between the parameter estimates.

**Results:**

The total sample size was 482 respondents, of which 207 (43%) lived in the United States, 181 (38%) lived in Kuwait, and 94 (20%) lived in South Korea. The ordered probit estimation results suggest that overall, perception of government response efforts positively influenced self-adherence (*P*<.001) and others’ adherence (*P*<.001) to social distancing and sheltering. Perception of government business reopening efforts positively influenced others’ adherence (*P*<.001). A higher intensity of general health information source for COVID-19 had a positive effect on self-adherence (*P*=.003). A higher intensity of social media source use for COVID-19 positively influenced others’ adherence (*P*=.002). A higher intensity of knowledge on COVID-19 positively influenced self-adherence (*P*=.008) and negatively influenced others’ adherence (*P*<.001). There were country-level variations—broadly, the United States and Kuwait had better effects than South Korea.

**Conclusions:**

As the COVID-19 global pandemic continues to grow and governmental restrictions are ongoing, it is critical to understand people’s frustration to reduce panic and promote social distancing to facilitate the control of the pandemic. This study finds that the government plays a central role in terms of adherence to restrictions. Governments need to enhance their efforts on publicizing information on the pandemic, as well as employ strategies for improved communication management to citizens through social media as well as mainstream information sources.

## Introduction

The coronavirus disease (COVID-19) has spread worldwide as an epidemic with serious implications [1]. Since its beginnings in Wuhan, China in December 2019, the COVID-19 pandemic has led to more than 5 million cases and 328,000 deaths reported across the world as of May 2020 [2]. Although around 2 million people have recovered from the virus, the prevalence is still high.

There is no known cure for COVID-19 as of now. Although vaccines are being tested, no established ones are widely available yet to prevent COVID-19 [3]. Infected individuals do not often exhibit any symptoms. The disease often progresses swiftly and kills patients at a much higher rate than the typical flu [4]. A few treatment options are possible for a patient with COVID-19. One of them is a ventilator that assists breathing. Because of limited testing and treatment availability, individuals must adopt preventive measures so that they do not get the virus [5]. Simple measures involve practicing good respiratory hygiene by washing hands with soap and water for at least 20 seconds at frequent intervals and avoiding touching the eyes, nose, or mouth with unwashed hands. More drastic efforts include social distancing, which is a set of nonpharmaceutical interventions or measures taken to prevent the spread of COVID-19 by maintaining a physical distance between people and reducing the number of times people come into close contact with each other; maintaining a safe distance from others; and sheltering practices by staying at home to avoid all contacts [3,6].

Governments in countries have instituted social distancing using policy directives and recommendations [7]. However, government oversights to influence citizens to social distancing and sheltering must wrestle with citizen’s willingness to comply or adhere to the recommendations relevant to the disease prevention and mitigation. For example, whether a citizen is willing to comply with the social distancing recommendations provided by the government and maintain a safe distance from others or stay at home. Another example is citizen adherence to wearing masks in public as a precaution against COVID-19 [8]. Significant variations are observed in citizens’ adherence to the COVID-19–relevant social distancing policy and masking in public across countries. The variation of agreeing that the guidelines are beneficial yet not following the guidelines by all citizens is a controversial issue in many countries [9,10]. A plausible reason for the variation in adherence to the policy recommendations by citizens is a culmination of informative and social influence [11] that is not explicitly understood in practice and academics well enough.

This study asks the research questions to what extent citizen’s perceptions about government efforts in the COVID-19 situation influence the adherence actions and to what extent information sources, social media (SM) use, and subsequent knowledge influence the adherence actions. Insights into the underlying factors and dynamics involved with citizens’ adherence process will inform the policy makers to follow appropriate communication and messaging strategies to influence citizens’ willingness to adhere to the recommendations.

Citizen’s perceptions of the government’s response to the COVID-19 situation and subsequent recovery efforts are critical in the context of implicit or explicit response to the social distancing recommendations. The adherence decision may be reliant on several factors. In the context of personal health compliance behavior, individuals follow health protection suggestions based on the perception of severity and vulnerability of a situation [12,13]. Given a health-relevant policy recommendation, individuals will also assess the efficacy of the recommendations and whether they have abilities to manage the process. Given that the COVID-19 context is a stressful situation, the compliance action will depend on the cognitive appraisal processes of the situation and the differing sensitivity, vulnerability, interpretations, and reactions to the situation [14,15]. Based on these arguments, we propose to test the conjectures: 

- Conjecture 1: Citizens’ perceptions of the government’s role to respond to the pandemic have a positive influence on adherence intention.
- Conjecture 2: Citizens’ perceptions of the government’s business reopening efforts have a positive influence on adherence intention.

The COVID-19 situation is not limited to *individuals* only; it extends to the social fabric. The policy recommendations have social implications as limiting interactions disrupts social group activities such as parties, get-togethers, and even shopping in malls. Thus, individual behavior would be affected by social norms and practices, as espoused by social psychology research [16,17], and individuals will try to understand and move through complex situations [18,19]. The COVID-19 situation is “difficult” in the sense that there is no treatment, and thus, individuals are more susceptible to learning from different sources and will subsequently manage the situation based on the knowledge gained from these information sources. The information available to people will also influence such behavior—more so in the current context of social media influences on people [20]. Therefore, we argue the variation in the adherence intentions at the individual level is an outcome of the information and knowledge gathering process. Thus, the common belief shaped by an individual is based on the information and knowledge that he gathers from different sources. Given that social media is emerging as a highly critical information source to influence individual beliefs and perceptions, it is beyond doubt that such an influence would be quite effective in the COVID-19 situation [20-22]. Thus, we propose to test the following conjectures: 

- Conjecture 3: A higher intensity of health information source (HIS) used for COVID-19 influences citizens’ adherence intentions.
- Conjecture 4: A higher intensity of social media source use for COVID-19 information influences citizens’ adherence intentions.
- Conjecture 5: A higher intensity of knowledge accrual for COVID-19 information influences citizens’ adherence intentions.

Although the individual level variations depend on the perceptions about the government actions and knowledge gained from different sources, the individual is also embedded within the normative influence of a country and its cultural fabric to follow certain social and cultural norms [23]. As already observed, governmental efforts toward the COVID-19 situation have differing outcomes across countries. To explore this normative influence, we conduct a comparative evaluation of citizens’ adherence process to COVID-19–relevant recommendations by the governments in three different countries: the United States, Kuwait, and South Korea. In summary, this study examines the impact of government efforts during the COVID-19 pandemic on citizens’ adherence, as well as the impact of information sources, social media use, and subsequent COVID-19 knowledge on citizens’ adherence in three different countries with differing cultures.

## Methods

### Recruitment

A focus group of 10 people in Kuwait was initially conducted. The focus group was asked to comment and provide insight into the subject matter. The feedback was that the cultural aspects are significant factors in terms of self adherence and belief of others adhering to governmental recommendations. Thus, we tried to expand to other countries with different cultures to understand the cultural impact on adherence better. However, although the chosen countries are different in culture, constrained by resources, the sampling strategy was limited to the countries that the authors are familiar with to gain firsthand experience to explain any similarities and differences.

A global survey-deploying firm collected the data for this study using online platforms. The firm recruited respondents from the United States, Kuwait, and South Korea in May 2020. The firm sampled respondents using age, gender, ethnicity, and a geographic region–based strata and quota matching process. Participation in the survey was free and voluntary; the respondents filled in electronic informed consent that was shown on the first page of the survey. The firm protects the confidentiality of anonymous respondents.

### Data Collection

Data was collected using a survey instrument, as shown in Multimedia Appendix 1 Table A1. The questions asked participants about the cause and current state of the COVID-19 situation, their opinion on the government’s role during the COVID-19 pandemic, and their information sources for the COVID-19 situation. The survey items included simple information-seeking questions, along with several existing validated scales from prior studies [24-30].

The survey instrument was pilot tested using a sample of 48 respondents, leading to minor refinements to a few items. A total of 535 participants took the survey. Because of missing responses to the items, 53 observations were excluded, resulting in a sample size of 482. Responses were coded, validated, and analyzed using STATA version 16 (StataCorp).

### Sample Demographics

Table 1 shows the descriptive statistics and pairwise correlations among the key variables used in this study. A detailed descriptive statistic, correlation tables, and distribution details of several demographic controls used in the models are available in Multimedia Appendix 1 (Table A2, Textbox A1, Table A5, and Figures A1-A5).

**Table 1:** Summary statistics and pairwise correlations among key variables (N=482).

Variable	Mean (SD)	Min	Max	1	2	3	4	5	6	7
Self-adherence (1)	4.28 (1.13)	1.00	5.00	1.00						
Other’s adherence (2)	3.16 (1.14)	1.00	5.00	0.24	1.00					
Response (3)	0.00 (0.80)	–2.66	0.75	0.69	0.31	1.00				
Reopen agreement (4)	2.34 (1.21)	1.00	5.00	–0.10	0.11	–0.22	1.00			
HIS^a^-general (5)	0.01 (0.56)	–1.80	2.14	0.11	0.02	0.00	0.13	1.00		
SM^b^-general (6)	–0.01 (0.92)	–2.58	1.95	–0.12	0.19	–0.03	0.01	–0.15	1.00	
Knowledge (7)	0.01 (2.33)	–7.37	5.53	0.34	–0.08	0.33	–0.20	–0.02	–0.11	1.00

^a^HIS: health information source.

^b^SM: social media.

### Study Variables

The main dependent variables in this study are self-adherence and others’ adherence. Self-adherence was measured using the question of whether they would comply with the social distancing measures. Others’ adherence was measured by asking whether the respondent believes that others would comply with social distancing measures. Table 1 shows that, on average, self-adherence was 4.28 out of 5, showing that most people are adhering to social distancing recommendations. However, the mean for others’ adherence was less, 3.16, showing lower levels of others’ adherence to social distancing.

Five independent variables were of interest in this study. First, the independent variable response reflects on the individual’s perception that the government response to the COVID-19 pandemic is effective. This variable was operationalized using four questions on the effectiveness, rights, and responsibilities of the government to respond to the COVID-19 situation (see Multimedia Appendix 1 Table A1). The internal consistency of the items was tested using Cronbach α (.81), and the standardized score was generated for the response variable. As Table 1 shows, the variable response has a mean of 0.00 and a standard deviation of 0.80.

The second independent variable, reopen agreement, reflects the individual’s perception of whether the government has a right to decide when to reopen businesses. The variable was measured with a single item on a five-point scale. The mean of this variable was 2.34, with 1.21 standard deviation, indicating the disagreement bias of respondents toward government plans to reopen businesses.

The respondents were asked where they obtained health information on COVID-19, whether from social media, TV, newspapers, friends and family, or doctors and health care specialists. The HIS-general variable was coded using the scheme mentioned in Multimedia Appendix 1 Table A1. Furthermore, the SM-general variable was coded to reflect on the individual’s intensity of social media platform use (further details on HIS-general and SM-general are provided in Multimedia Appendix 1 Textbox A2, Table A3, and Table A4).

The knowledge variable was coded to reflect the respondents’ overall knowledge of COVID-19, as mentioned in Multimedia Appendix 1 Table A1. The mean for knowledge was 0.01, with a minimum of –7.37 and a maximum of 5.53, showing poor overall knowledge on COVID-19.

In addition to these key variables of interest, several control variables, as mentioned in Multimedia Appendix 1 Table A1, are included to account for counterfactual explanations relevant to our models.

### Econometric Analysis

The empirical model specifies how individuals express their opinion toward adherence to social distancing and sheltering guidance set by the government. We specified self-adherence and others’ adherence as two dependent variables. The set of independent variables we focused on include response, reopen agreement, HIS-general, SM-general, and knowledge. A set of control variables to enhance the robustness of our empirical model include demographics characteristics of the survey participants, such as gender, age group, household income, and ethnicity. The empirical model is described below:

*Y(Self-Adherence, Others’ Adherence)_i_ = β_0_ + β_1_ × Response_i_ + β_2_ × Reopen Agreement_i_ + β_3_ × HIS-General_i_ + β_4_ × SM-General_i_ + β_5_ × Knowledge_i_ + Controls_i_ + ε_i_*

where *Controls* include gender, age groups, household income, and ethnicity. The country dummy was included in the full sample model but was removed for subsample analyses. Since the dependent variables are ordinal values, we used an ordered probit model to estimate the parameters of the key variables with robust standard errors (see Multimedia Appendix 1 Textbox A3 for details on the model). Using ordered probit regression, we estimated to what extent our set of key variables influence self-adherence and others’ adherence separately.

## Results

Table 2 presents the key estimation results (detailed estimations are provided in Multimedia Appendix 1 Table A6). The first four columns (1-4) in the table show the parameter estimates for the self-adherence dependent variable for the United States, Kuwait, South Korea, and the full sample (all). The columns 5-8 in Table 2 show the parameter estimates for the others’ adherence regression for the United States, Kuwait, South Korea, and the full sample (all).

The first set of findings are relevant to the effect of the response variable on the outcomes. We found that the coefficients of response were positive and statistically significant at *P*<.001 across all columns. This suggests that individuals who believe that government response efforts are positive follow adherence guidance on social distancing and sheltering. At the same time, higher perception that government response efforts are positive leads individuals to believe that others will also follow adherence guidance on social distancing and sheltering.

We compared the coefficients of the estimated coefficients using a model-based chi-square comparison test with Bonferroni adjustments. Table 3 presents the results. We found that the positive effect of response on adherence shows that individuals who reside in the United States and Kuwait are more likely to follow adherence than South Korea.

**Table 2:** Ordered probit regression.^a^

Variables	DV^b^: self-adherence								DV: others’ adherence							
	(1)		(2)		(3)		(4)		(5)		(6)		(7)		(8)	
	US	*P* value	Kuwait	*P* value	South Korea	*P* value	All	*P* value	US	*P* value	Kuwait	*P* value	South Korea	*P* value	All	*P* value
Response	1.192 (0.154)^c^	<.001	0.929 (0.205)	<.001	2.342 (0.279)	<.001	1.108 (0.097)	<.001	0.353 (0.124)	.003	0.520 (0.133)	.001	1.494 (0.174)	<.001	0.636 (0.076)	<.001
Reopen agreement	–0.021 (0.083)	.82	–0.109 (0.106)	.33	–0.091 (0.120)	.53	–0.008 (0.054)	.89	0.106 (0.081)	.14	0.233 (0.088)	.004	0.381 (0.148)	.005	0.174 (0.050)	.001
HIS^d^-general	0.356 (0.174)	.06	0.532 (0.211)	.02	0.697 (0.289)	.049	0.309 (0.105)	.003	0.200 (0.114)	.17	–0.149 (0.131)	.33	0.537 (0.401)	.03	0.009 (0.089)	.92
SM^e^-general	0.010 (0.124)	.94	–0.132 (0.137)	.45	0.209 (0.274)	.51	0.002 (0.073)	.98	0.238 (0.113)	.03	0.329 (0.124)	.006	0.568 (0.287)	.05	0.254 (0.068)	<.001
Knowledge	0.121 (0.049)	.02	–0.041 (0.073)	.53	0.114 (0.088)	.18	0.074 (0.028)	.008	–0.009 (0.044)	.82	–0.155 (0.050)	.001	–0.054 (0.072)	.45	–0.086 (0.024)	<.001
Observations, n	207	N/A^f^	181	N/A	94	N/A	482	N/A	207	N/A	181	N/A	94	N/A	482	N/A
Pseudo *R*^2^	0.211	N/A	0.222	N/A	0.505	N/A	0.268	N/A	0.054	N/A	0.134	N/A	0.328	N/A	0.092	N/A
χ^2^ (*df*)	84.22 (21)	N/A	48.437 (21)	N/A	115.427 (21)	N/A	219.146 (21)	N/A	30.05 (21)	N/A	108.713 (21)	N/A	132.198 (21)	N/A	108.604 (21)	N/A

^a^Models include all controls: age, gender, household income, ethnicity.

^b^DV: dependent variable.

^c^Standard errors in parentheses.

^d^HIS: health information source.

^e^SM: social media.

^f^N/A: not applicable.

**Table 3:** Comparison of coefficients across countries.^a^

Variables	DV^b^: self-adherence						DV: others’ adherence					
	US vs Kuwait	*P* value	US vs South Korea	*P* value	Kuwait vs South Korea	*P* value	US vs Kuwait	*P* value	US vs South Korea	*P* value	Kuwait vs South Korea	*P* value
Response	1.05	.84	13.09	.002	16.73	.01	0.85	>.99	28.68	<.001	19.83	<.001
Reopen agreement	0.43	.97	0.23	.99	0.01	>.99	1.13	>.99	2.68	.51	0.74	>.99
HIS^c^-general	0.42	.97	1.03	.84	0.21	.99	4.03	.22	0.66	>.99	2.66	.52
SM^d^-general	0.60	.95	0.44	.97	1.25	.78	0.30	>.99	1.16	>.99	0.59	>.99
Knowledge	3.39	.29	0.00	>.99	1.85	.62	4.78	.14	0.29	>.99	1.31	>.99
All countries	6.09	.29	13.36	.02	20.15	.001	10.14	.07	33.52	<.001	30.55	<.001

^a^Chi-square values reported with Bonferroni adjustment.

^b^DV: dependent variable.

^c^HIS: health information source.

^d^SM: social media.

The second set of findings were related to the effect of reopen agreement on the outcomes. As shown in Table 2, we found that the coefficient of reopen agreement was not significant for self-adherence for all the models. However, the coefficients of reopen agreement were positive and statistically significant for others’ adherence for Kuwait (*P*=.008) and South Korea (*P*=.01) but not significant for the United States (*P*=.19). This set of findings suggests that in Kuwait and South Korea, an individual’s opinion on the government’s reopening efforts influence only their belief on others’ following adherence guidance on social distancing and sheltering practices. In contrast, in the case of the United States, the agreement on the reopening of businesses does not have any effect on any adherence outcomes. Interestingly, across countries, there is not a significant comparative difference in this effect, as the comparative chi-square values are not significant, as shown in Table 3.

A third finding was that the coefficients of HIS-general were positive and statistically significant (*P*=.003) for self-adherence only and not on others’ adherence, suggesting that individuals use various information sources to guide their actions on adherence but not on others. Fourth, the coefficients of SM-general were positive and statistically significant for the United States (*P*=.04), South Korea (*P*=.048), and Kuwait (*P*=.008) for others’ adherence only, suggesting that individuals use social media to formulate their belief on others’ adherence to social distancing and sheltering behavior. Comparisons of coefficients for HIS-general and SM-general across three countries did not show statistically significant results.

Finally, the coefficients of knowledge on COVID-19 show different effects on self-adherence and others’ adherence. For US residents, knowing more about the virus is positively associated with self-adherence (column 1 of Table 2). However, residents in Kuwait who know more about the virus are less likely to believe that others will adhere to social distancing and sheltering guidance (column 6 of Table 2). The full sample analysis shows that the direction of the coefficients was positive for self-adherence and negative for other’s adherence, suggesting that this effect is consistent overall but differs across countries.

## Discussion

### Findings and Implications

In general, this study finds that, in all three countries, government response efforts, business reopening agreements, as well as the intensity of information source use, social media use, and knowledge about the COVID-19 pandemic all influence either self-adherence or belief in others adhering. Table 4 summarizes the findings from this study.

**Table 4:** Summary of findings.

Variables	Self-adherence				Others’ adherence				Findings
	US	Kuwait	South Korea	All	US	Kuwait	South Korea	All	
Response	Pos^a^	Pos	Pos	Pos	Pos	Pos	Pos	Pos	C^b^1: Supported for self-adherence and others’ adherence; US and Kuwait have better outcomes than South Korea
Reopen agreement	NS^c^	NS	NS	NS	NS	Pos	Pos	Pos	C2: Partially supported for others’ adherence; no comparative difference results across countries
HIS^d^-general	Pos	Pos	Pos	Pos	NS	NS	NS	NS	C3: Supported for self-adherence; no comparative difference results across countries
SM^e^-general	NS	NS	NS	NS	Pos	Pos	Pos	Pos	C4: Supported for others’ adherence; no comparative difference results across countries
Knowledge	Pos	NS	NS	Pos	NS	Neg^f^	NS	Neg	C5: Partially supported for self-adherence (positive for the US only) and others’ adherence (negative for Kuwait only)

^a^POS: positive association.

^b^C: conjecture.

^c^NS: not significant.

^d^HIS: health information source.

^e^SM: social media.

^f^Neg: negative association.

The first finding inherently suggests a *persuasion effect* for governments’ adherence guidelines on individuals. This persuasion effect in terms of conveying information and communicating risks to the public during the COVID-19 crisis are becoming complicated due to the large amount of misinformation, uncertainty surrounding the source and spread of the virus, and the absence of a vaccine [31-35]. However, previous research has shown that trusting the health system has a significant impact on public willingness to receive and adhere to health instructions [36]. This persuasion effect is highly effective when the government provides guidelines’ context (ie, detailed explanations, implications, and consequences of the guidelines to the individuals). However, the magnitude of the persuasion effect differs across the three countries sampled in this study. Several reasons can be attributed to explain the differences, with a plausible one pointing to the expectations of citizens that the government is doing everything right for them. In the case of the United States and Kuwait, the citizens expect that the governments need to provide enough information and rationale to take away their individual and social freedom in the form of sheltering and distancing measures. However, citizens of South Korea are more willing to follow government guidelines during a national crisis.

The second finding suggests that individuals expect that others will adhere to guidelines given that they agree to the reopening stipulations—although they themselves may not do so. A plausible argument points to human’s self-interests. To instantiate, individuals would expect that life should go back to normal in the post–COVID-19 situation [37]. The COVID-19 situation has led to countrywide unemployment, economic downturn, and a lack of supplies—leading to a stressful situation for individuals who carry on with their livelihoods. Although reopening provides avenues for economic recovery, there is no guarantee that people will follow the suggested safe-distancing and other guidelines. Research finds that there is a positive and significant correlation between overall risk perception and economic threat perception [36]. People that perceive a high personal economic threat may feel that the government is not managing the crisis well; this thereby impacts their adherence. Therefore, individuals are a bit cautious but hopeful that recovery attempts will increase others’ adherence to the guidelines.

The third set of findings emphasizes the information-based decision-making process of individuals in the COVID-19 situation. As much as we have validated the influence of the perceptions of the individuals on the government’s actions, the adherence decision is influenced by the information that is available to the individual. Moreover, following our coding scheme and the sensitivity checks of the impact of general versus specific information sources on the self-adherence outcomes, it can be inferred that channeling information to citizens should follow a broader spectrum [34]. In other words, individuals are influenced by broader information sources such as friends, families, doctors, and social sources rather than relying entirely on only popular channels such as newspapers and television. This is the same irrespective of the individual’s country, referring to any lack of comparative effects across the countries. This finding is in line with research done on the social influence that shows that health behaviors spread through perceived norms with whom common identities are most shared [38]. Social networks can amplify the spread of behavior regardless of whether the behavior is harmful or beneficial to the pandemic [39,40]. Therefore, sending out information such as *nudges* [40] that is related to what others within the community are doing would influence an individual adherence level. Nudges and normative information may be an alternative method that the government can use to impose the spread of policies better.

The fourth set of findings highlights some interesting roles of social media on the individual’s adherence decisions in the COVID-19 situation. Although previous studies have found an impact of social media use on individual behavior during the COVID-19 pandemic [41,42], this study examines the impact of social media use on adherence. This study finds that the influence of social media is significant on others’ adherence but not on self-adherence. We contrasted and compared this with the previously mentioned information source effects. As it was observed, information sources influence self-adherence, but social media influences others’ adherence. These findings would suggest that shaping social norms is effective through social media. In this effect, the prominence of social media platforms such as Twitter, Instagram, Snapchat, and WhatsApp is additionally validated in the analysis, compared to Facebook and YouTube. Plausibly, some social media platforms such as Twitter and Instagram are relatively informative; some are more socially inclusive ones; WhatsApp, Snapchat, and a few others like YouTube and Facebook are entertainment-oriented platforms. These distinctions are not exclusive but may help to explain the higher impact of informative social media versus entertaining social media to shape an expected social norm regarding adhering decisions.

The final set of findings and implications are about the effect of the knowledge level on the symptoms, treatments, and risks associated with COVID-19 on adherence intentions. A recent study has found a positive and significant correlation between awareness and knowledge, and adherence to social distancing [43]. This study supports this finding and further finds that a higher knowledge level is influential in shaping the decisions on self-adherence for the United States, whereas it raises a skepticism on others’ adherence decisions for Kuwait. A possible reason may be due to differences in the cultural norm as well as the information surrounding the culture or community. Disseminating accurate information about what most people are doing is helpful and health-promoting; however, if the surrounding community is not health-promoting, providing purely descriptive normative information may backfire by reducing positive behaviors among people who already engage in them [40]. Furthermore, when it comes to health information dissemination, often less is better than giving too much, as deeper knowledge may sway different perceptions or lead to anxiety and panic [44,45]. Thus, a balanced approach in disseminating details about COVID-19 and possible implications is essential while keeping in mind that not all information would lead to positive outcomes.

### Practice and Policy Implications

A set of implications and recommendations for public health officials and policy makers can be drawn from this study. This study points to the gap in the responsible behavior of individuals while following self-adherence versus expecting others’ decisions. In that, the current government efforts to mitigate and the expectation that everything should be back to normal have differing consequences. Existing work suggests that the efficacy and outcomes of policy recommendations depend on the individuals’ beliefs and subsequent action [46,47]. The first step in this process is the strong belief of whether the recommended action will help in mitigating the threat or situation. The key to minimizing rejection and maximizing acceptance of recommendation, thus, is reliant on how the recommendations are framed and the subsequent ways the relevant information is disseminated and communicated. Care needs to be taken in framing information dissemination and communication channels in managing the current COVID-19 pandemic.

The understanding of the aspects relevant to the persuasion process of information dissemination to citizens so that not only negative and fear-based appeals and messages are spread but also positive and emotion-based messages that can lead to better appeals is important. As it is already seen, popular press and television channels may not do justice to the messages unless the source design of the messages constructs the message appropriately. The snowballing effects of fear-based appeals and subsequent rejection would have a highly negative consequence.

The study also highlights the importance of information sources in terms of compliance intention and, thus, the importance of managing misinformation and misleading channels. Health authorities have warned that widespread misinformation about COVID-19 is a severe concern causing xenophobia worldwide [48]. Given the spread of information through the channels, it is not easy for individuals to discern individual responsibilities and expectations of others, without which the situation may get quickly aggravated and spread panic. Thus, as much as channels are used to disseminate, the prominence of social media in this pandemic cannot be underestimated [40], and effective social media strategies need to be deployed by policy makers to defuse the fear, anxiety, and panic-inducing effects of information by nudging positive health behaviors within communities [14,49].

This study has implications for health systems across countries that are facing heightened levels of stress because of COVID-19. Efforts to contain the spread of the disease have virtually failed, leading to ventilator-based treatment of patients with COVID-19. Health systems are working to increase supply by ﬁnding any way possible to create capacity for patients with COVID-19 while following tactics such as stopping elective procedures and surgeries.

In this context, knowledge-based decisions by citizens play an important role, such as to know whether to go to a hospital for COVID-19 symptoms or not. Knowledge of exact symptoms and risks may empower citizens to make decisions at their ends and stay away from hospitals, and avail possible treatments at their end.

Thus, this study unravels a set of insights, reflecting and collecting data on the differing adherence outcomes across countries. The insights inform four policy recommendations: (1) governments should tell citizens precisely what individuals need to do for themselves; (2) governments should also tell what individuals should expect from others and what others need to do when dealing with other individuals; (3) messages from policy makers to citizens must be done through social media and similar channels rather than only through press and TV; and (4) messages need to provide knowledge about the disease along with information that should calm the citizens, and messages should nudge citizens on what others in their community are doing in terms of health-promoting behavior.

### Limitations

This study examines factors that influence citizens adhering to social distancing and their belief in others’ adherence of social distancing at a point in time. However, the citizen might go back and forth in the adherence process, and the knowledge level or source of information may change over time. This is a limitation of this study, as the data set used is a cross-sectional survey. Future studies could examine how a citizen’s adherence changes over time. In addition, using the random sampling process of the public in the United States, Kuwait, and South Korea samples may include fewer familiar respondents to the study context. In particular, the questionnaire was on the internet. Therefore, respondents are all users of the internet. The study does not examine noninternet users, which could have differential impacts. Thus, the generalization of the sample to a uniform national culture characteristic is a limitation of this study. Future studies could conduct both internet and noninternet surveys, and examine the difference in terms of adherence behavior.

### Conclusions

The rapid spread of COVID-19 has generated great public interest and media coverage around the outbreak, and it has created social unrest and economic downturn. Strict measures of mitigation have been implemented to avoid the breakdown of the health system [50] and reduce the deaths caused by the virus. Efforts to contain the spread of the disease have virtually failed with efforts to find a way to keep the economies sustained. In this context, the social distancing policy deems to be a lifesaver not only for individuals but also for economies across the world.

As the COVID-19 global pandemic continues to grow and governmental restrictions are ongoing, it is critical to understand people’s frustration to reduce panic and promote social distancing to facilitate the control of the pandemic. This study finds that the government plays a central role in terms of adherence to restrictions and provides several insights that are important for the government to frame the message, information, and communication for the citizens.

The pandemic may end socially or medically—in the sense that, prior to the vaccine or effective treatment, exhausted and frustrated people will return to regular life [51]. As several countries are lifting restrictions, listening to people’s voices of frustrations, an equal and opposite criticism is often voicing that these steps are premature. This conflict is not easy to resolve and points to the need for understanding public voices and opinions more carefully than the medical end of a pandemic. This study is a step in exploring more nuances to the citizen’s mindsets underlying the voices, and we expect that the recommendations based on the insights will provide effectiveness to the social distancing and masking measures, and will help to curb COVID-19.

## Appendix

Multimedia Appendix 1 Survey questionnaire and further detailed analysis.

## Abbreviations

COVID-19: coronavirus disease
HIS: health information source
SM: social media
