# Supplementary material for: Citizens’ Adherence to COVID-19 Mitigation Recommendations by the Government: A 3-Country Comparative Evaluation Using Web-Based Cross-Sectional Survey Data
Source: J Med Internet Res. 2020 Aug 11;22(8):e20634. doi: 10.2196/20634 (PMC7423388; doi:10.2196/20634)
Supplement: Multimedia Appendix 1 [file jmir_v22i8e20634_app1.doc]

**Multimedia Appendix 1 : Survey questionnaire and further detailed analysis.**

Table A1: Survey Questionnaire and Coding Scheme

| **Variable** | **Description** | **Questionnaire Items***.* | **Refs.** |
| --- | --- | --- | --- |
| *Dependent Variables* | | | |
| Y1:  SELF-ADHERENCE | Individual’s intention to adhere to social distancing/sheltering recommendations | - I will comply with the sheltering or social distancing measures   *Scale: 1 - Strongly Disagree to 5-Strongly Agree* | [24-29] |
| Y2:  OTHERS’-ADHERENCE | Whether others will adhere to social distancing/sheltering recommendations, as perceived by the individual | - Others will comply with the sheltering or social distancing measures   *Scale: 1 - Strongly Disagree to 5-Strongly Agree* | [24-29] |
| *Independent Variables* | | | |
| X1:  RESPONSE | The perception that the government’s response to COVID-19 situation is effective | - Sheltering or social distancing measures are effective at slowing the spread of Covid-19 - The government has the right to enforce sheltering (i.e., people must stay at home) - The government has the right to use technology to track people for mitigating the spread of Covid-19 - The government has the responsibility to inform its citizens the potential for a pandemic like Covid-19   *Scale: 1 - Strongly Disagree to 5-Strongly Agree.*  *Cronbach Alpha: 0.81.* | [24-29] |
| X2:  REOPEN-AGREEMENT | The perception that the government does not have the right to decide when to reopen businesses. | - The government does not have the right to decide when to reopen businesses   *Scale: 1 - Strongly Disagree to 5-Strongly Agree* | [24-29] |
| X3:  HIS-GENERAL | This variable reflects the intensity of general information sources that the individual uses to gather or collect information about the COVID-19 situation. | - Which of the following do you use for Covid-19 related information? (a) Social Media, (b) TV, (c) Newspaper (online and print), (d) Friends or Family, (e) Doctors or Medical Professionals   *Scale: 1 - Yes, 0 – No.*  *An exploratory factor analysis of the yes responses generated two factors,* HIS-GENERAL and HIS-REPUTABLE*. HIS here indicates health information sources. The HIS-GENERAL is a focal independent variable, whereas the other two variables are used as controls in the estimation models.* |  |
| X4:  SM-GENERAL | The intensity of general social media (SM) sources that the individual uses to gather or collect information about COVID-19 situation. | - Which of the following social media do you use for Covid-19 news? (a) Facebook, (b) Twitter, (c) WhatsApp, (d) Instagram, (e) LinkedIn, (f) Snapchat, (g) TikTok, (h) YouTube, (i) Other.   *Scale: 1 - Yes, 0 – No.*  *An exploratory factor analysis of the yes responses generated three factors, and those are coded as* SM-GENERAL, SM-ENTERTAIN*, and* SM-WORK *variables. The* SM-GENERAL *is the focal independent variable, whereas the other two variables are used as controls in the estimation models.* |  |
| X5:  KNOWLEDGE | The extent to which one is aware or knowledgeable about COVID-19 and relevant situations. | - What are the symptoms of Covid-19? (a) Fever, (b) Cough, (c) Difficulty breathing, (d) Nausea, (e) Diarrhea, (f) Skin Rash, (g) Loss of smell and taste, (h) I don’t know - Which of the following characteristics are associated with a high-risk group? (a) Elderly, (b) Diabetes, (c) Smokers, (d) Female, (e) Other - What is the treatment of Covid-19? (a) Drink fluid and getting rest, (b) Hydroxychloroquine, (c) Herbal medicine, (d) Vaccines, (e) None   *Scale: 1 - Yes, 0 – No.*  *The aggregated and standardized items* *were taken to code the* KNOWLEDGE *variable.* | [30] |
| X6:  COUNTRY | Country of residence | - I reside in: (a) United States, (b) South Korea, (c) Kuwait.   *Dummy variables were coded for countries based on responses.* |  |
| *Control Variables* | | | |
| C1:  AGE | Age of respondent | My age group is: (a) 18-27 years old, (b) 28-37 years old, (c) 38-47 years old, (d) 48-57 years old, (e) greater than 58 years old | |
| C2:  GENDER | Gender of respondent | I am: (a) Male, (b) Female | |
| C3:  INCOME | Household income of the respondent | My household income is: (a) Less than $30,000, (b) $30,000 - $50,000, (c) $50,000 - $80,000, (d) $80,000 - $100,000, (e) $100,000 - $150,000, (f) Higher than $150,000 | |
| C4:  ETHNICITY | Ethnicity of respondent | My ethnicity is: (a) White, (b) Middle Eastern, (c) Asian or Pacific Islander, (d) Others (including Black of African descent, Latino, and Mixed). | |

Table A2. Summary Statistics of Key Variables (N=482)

| **Variable** | **Mean** | **Std. Dev.** | **Min** | **Max** |
| --- | --- | --- | --- | --- |
| SELF-ADHERENCE | 4.28 | 1.13 | 1 | 5 |
| OTHERS’-ADHERENCE | 3.16 | 1.14 | 1 | 5 |
| RESPONSE | 0.00 | 0.80 | -2.66 | 0.75 |
| REOPEN AGREEMENT | 2.34 | 1.21 | 1 | 5 |
| HIS-GENERAL | 0.01 | 0.56 | -1.80 | 2.14 |
| HIS-REPUTABLE | 0.00 | 0.38 | -1.14 | 1.21 |
| SM-GENERAL | -0.01 | 0.92 | -2.58 | 1.95 |
| SM-ENTERTAIN | -0.01 | 0.58 | -1.47 | 2.08 |
| SM-WORK | 0.00 | 0.40 | -1.23 | 1.12 |
| KNOWLEDGE | 0.01 | 2.33 | -7.37 | 5.53 |
| AGE GROUP - 18-27 | 0.38 | 0.49 | 0 | 1 |
| AGE GROUP - 28-37 | 0.30 | 0.46 | 0 | 1 |
| AGE GROUP - 38-47 | 0.13 | 0.34 | 0 | 1 |
| AGE GROUP - 48-57 | 0.08 | 0.27 | 0 | 1 |
| AGE GROUP - 58+ | 0.11 | 0.32 | 0 | 1 |
| FEMALE | 0.58 | 0.49 | 0 | 1 |
| HOUSEHOLD INCOME - Less than $30K | 0.20 | 0.40 | 0 | 1 |
| HOUSEHOLD INCOME - $30K-$50K | 0.18 | 0.39 | 0 | 1 |
| HOUSEHOLD INCOME - $50K - $80K | 0.21 | 0.40 | 0 | 1 |
| HOUSEHOLD INCOME - $80K-$100K | 0.13 | 0.34 | 0 | 1 |
| HOUSEHOLD INCOME - $100K-$150K | 0.11 | 0.32 | 0 | 1 |
| HOUSEHOLD INCOME - $150K or more | 0.16 | 0.37 | 0 | 1 |
| ETHNICITY – WHITE | 0.46 | 0.50 | 0 | 1 |
| ETHNICITY – ASIAN | 0.24 | 0.43 | 0 | 1 |
| ETHNICITY – OTHER | 0.16 | 0.37 | 0 | 1 |
| ETHNICITY - MIDDLE EASTERN | 0.14 | 0.35 | 0 | 1 |
| COUNTRY – SOUTH KOREA | 0.20 | 0.40 | 0 | 1 |
| COUNTRY – US | 0.43 | 0.50 | 0 | 1 |
| COUNTRY – KUWAIT | 0.38 | 0.48 | 0 | 1 |

Textbox A1. Demographics Distribution Details of Sample

| Of the 482 survey participants in this study, 280 (58%) identified themselves as female. In terms of age group distribution, 183 participants (38%) were in the 18-27 years of age group, 144 (30%) in the 28-37 years of age group, 63 (13%) in the 38-47 years of age group, 37 (8%) in the 48-57 years of age group, and 55 (11%) in the 58 years or above age group. Figure A1 shows the wise country comparison of the age of respondents.  In terms of income level, 98 participants (20%) reported they earned less than $30,000 annually, 89 (18%) earned between $30,000-$50,000, 99 (21%) earned between $50,000 - $80,000, 64 (13%) earned between $80,000 - $100,000, 54 (11%) earned between $100,000 - $150,000, and 78 (16%) earned more than $150,000. This distribution of household income also varies by country as shown in Figure A2.  In terms of ethnicity distribution, 220 participants (46%) reported their ethnicity as White (46%), 115 (24%) as Asian, 69 (14%) as Middle Eastern, and 78 (16%) reported their ethnicity as Black, mixed, and other. In terms of place of residence, 207 (43%) lived in US, 181 (38%) lived in Kuwait, and 94 (20%) lived in South Korea (Figure A3). |
| --- |


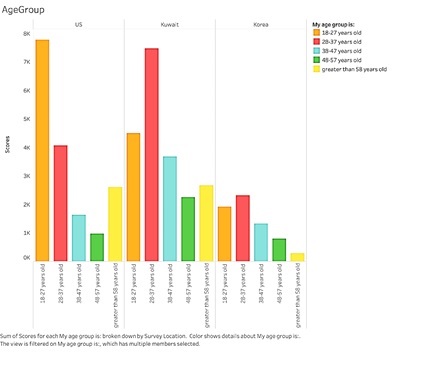


Figure A1. Age groups distribution by country.

**
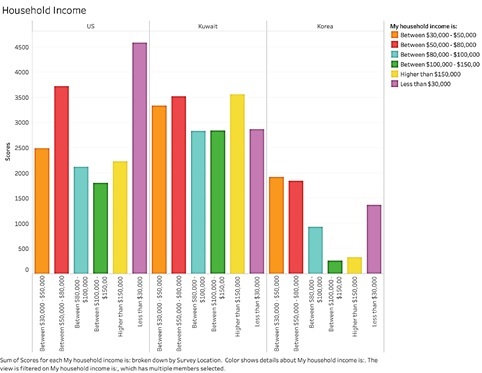
**

Figure A2. Household income distribution by country.

**
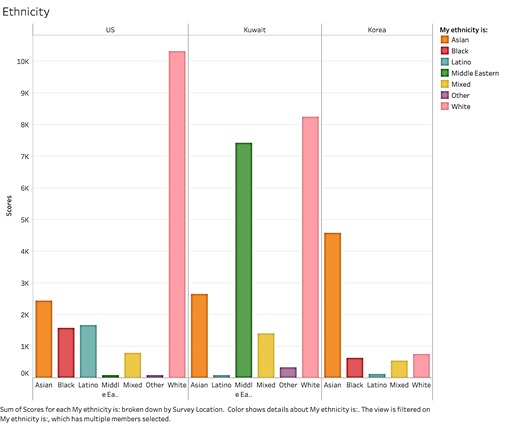
**

Figure A3. Ethnicity distribution by country.

**
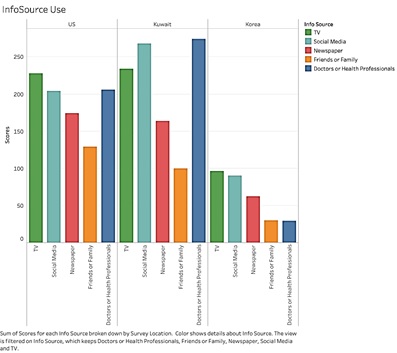
**

Figure A4. Information source for Covid-19 by country.

**
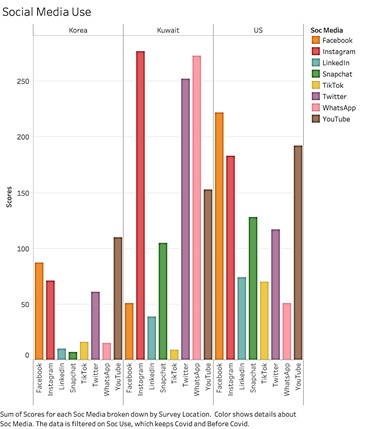
**

Figure A5. Social media use for Covid-19 by country.

Textbox A2. Details on Health Information Sources

| The respondents were asked where they obtained HEALTH INFORMATION SOURCE on COVID-19, whether from social media, TV, newspapers, friends/family, or doctors and healthcare specialists. A single multi-item questionnaire asked participants to select one or more information sources for health information on COVID-19. As shown in Figures A4 and A5, the responses were coded as a dummy variable for each type of health information source. The coded responses were transformed into two factors using factor analysis: (1) General and (2) Reputable health information. Furthermore, participants were also asked what SOCIAL MEDIA sources were used to attain COVID-19 information. The multi-item questionnaire consisted of eight popular social media platforms such as Facebook, Twitter, Instagram, Snapchat, WhatsApp, LinkedIn, TikTok, and YouTube. Operationalization of the coded item was carried out similarly to that of HEALTH INFORMATION SOURCE, and three factors have been identified – (1) General, (2) Entertainment, and (3) Work-related social media use. Table A2 and Table A3 show the factor loadings for HEALTH INFORMATION SOURCE and SOCIAL MEDIA use, respectively. |
| --- |

Table A3. Factor Loadings of HEALTH INFORMATION SOURCE

| Variable | Factor1 | Factor2 | Uniqueness |
| --- | --- | --- | --- |
| Social Media | 0.487 | -0.1896 | 0.7268 |
| TV | 0.4579 | 0.1357 | 0.7719 |
| Newspaper | 0.4609 | 0.1748 | 0.757 |
| Friends/Family | 0.4909 | -0.1739 | 0.7287 |
| Doctors | 0.2736 | 0.1281 | 0.9087 |

Table A4. Factor Loadings of SOCIAL MEDIA use

| Variable | Factor1 | Factor2 | Factor3 | Uniqueness |
| --- | --- | --- | --- | --- |
| Facebook | 0.098 | 0.445 | -0.044 | 0.790 |
| Twitter | 0.378 | -0.126 | 0.059 | 0.838 |
| Instagram | 0.486 | -0.063 | -0.083 | 0.753 |
| Snapchat | 0.461 | -0.061 | 0.045 | 0.782 |
| WhatsApp | 0.445 | -0.264 | -0.037 | 0.732 |
| LinkedIn | 0.162 | 0.162 | 0.155 | 0.924 |
| TikTok | 0.161 | 0.251 | -0.023 | 0.911 |
| YouTube | 0.298 | 0.380 | -0.012 | 0.767 |

Table A5. Pairwise correlation amongst key variables

| Variables | (1) | (2) | (3) | (4) | (5) | (6) | (7) | (8) | (9) | (10) |
| --- | --- | --- | --- | --- | --- | --- | --- | --- | --- | --- |
| 1. SELF-ADHERENCE | 1.00 |  |  |  |  |  |  |  |  |  |
| 1. OTHER-ADHERENCE | 0.24 | 1.00 |  |  |  |  |  |  |  |  |
| 1. RESPONSE | 0.69 | 0.31 | 1.00 |  |  |  |  |  |  |  |
| 1. RECOVERY | -0.10 | 0.11 | -0.22 | 1.00 |  |  |  |  |  |  |
| 1. HIS-GENERAL | 0.11 | 0.02 | 0.00 | 0.13 | 1.00 |  |  |  |  |  |
| 1. HIS-REPUTABLE | 0.03 | -0.01 | -0.06 | 0.06 | -0.04 | 1.00 |  |  |  |  |
| 1. SM-GENERAL | -0.12 | 0.19 | -0.03 | 0.01 | -0.15 | -0.06 | 1.00 |  |  |  |
| 1. SM-ENTERTAIN | -0.03 | 0.10 | 0.05 | -0.04 | -0.08 | -0.09 | 0.38 | 1.00 |  |  |
| 1. SM-WORK | -0.05 | -0.06 | -0.08 | 0.04 | 0.12 | 0.08 | -0.20 | -0.17 | 1.00 |  |
| (10) KNOWLEDGE | 0.34 | -0.08 | 0.33 | -0.20 | -0.02 | 0.02 | -0.11 | 0.02 | 0.00 | 1.00 |

Table A6. Detailed Estimation Results

|  | SELF-ADHERENCE | | | OTHER-ADHERENCE | | | FULL SAMPLE | |
| --- | --- | --- | --- | --- | --- | --- | --- | --- |
|  | (1) | (2) | (3) | (4) | (5) | (6) | (7) | (8) |
| Variables | US | KUWAIT | SOUTH  KOREA | US | KUWAIT | SOUTH  KOREA | SELF | OTHER |
| Main Independent Variables | | | | | | | | |
| RESPONSE | 1.192^***^  (0.154) | 0.929^***^  (0.205) | 2.342^***^  (0.279) | 0.353^**^  (0.124) | 0.520^***^  (0.133) | 1.494^***^  (0.174) | 1.108^***^  (0.097) | 0.636^***^  (0.076) |
| RECOVERY | -0.021  (0.083) | -0.109  (0.106) | -0.091  (0.120) | 0.106  (0.081) | 0.233^**^  (0.088) | 0.381^**^  (0.148) | -0.008  (0.054) | 0.174^***^  (0.050) |
| HIS-GENERAL | 0.356^*^  (0.174) | 0.532^*^  (0.211) | 0.697^*^  (0.289) | 0.200  (0.114) | -0.149  (0.131) | 0.537  (0.401) | 0.309^**^  (0.105) | 0.009  (0.089) |
| HIS-REPUTABLE | 0.759^**^  (0.284) | 0.179  (0.343) | 0.241  (0.279) | 0.193  (0.204) | 0.056  (0.252) | 0.104  (0.347) | 0.318^*^  (0.149) | 0.061  (0.129) |
| SM-GENERAL | 0.010  (0.124) | -0.132  (0.137) | 0.209  (0.274) | 0.238^*^  (0.113) | 0.329^**^  (0.124) | 0.568^*^  (0.287) | 0.002  (0.073) | 0.254^***^  (0.068) |
| SM-ENTERTAIN | -0.087  (0.191) | -0.132  (0.249) | -0.082  (0.252) | 0.211  (0.160) | 0.037  (0.169) | 0.098  (0.229) | -0.075  (0.112) | 0.071  (0.094) |
| SM-WORK | -0.541^*^  (0.229) | 0.162  (0.306) | -0.837  (0.571) | 0.077  (0.185) | -0.190  (0.254) | 0.610  (0.403) | -0.292^*^  (0.144) | -0.038  (0.135) |
| KNOWLEDGE | 0.121^*^  (0.049) | -0.041  (0.073) | 0.114  (0.088) | -0.009  (0.044) | -0.155^**^  (0.050) | -0.054  (0.072) | 0.074^**^  (0.028) | -0.086^***^  (0.024) |
| *Age Group (Base: under 28)* | | | | | | | | |
| Age 28-37 | -0.084  (0.242) | -0.341  (0.283) | 0.725  (0.460) | -0.128  (0.235) | -0.325  (0.210) | 0.648  (0.397) | -0.056  (0.154) | -0.283^*^  (0.137) |
| Age 38-47 | -0.915^**^  (0.339) | -0.482  (0.347) | 0.055  (0.472) | -0.786^*^  (0.313) | 0.728^**^  (0.255) | 0.669  (0.435) | -0.370^*^  (0.181) | 0.068  (0.180) |
| Age 48-57 | -1.418^*^  (0.571) | -0.123  (0.408) | 0.668  (0.503) | -0.775^*^  (0.343) | 0.236  (0.314) | 0.976^*^  (0.408) | -0.390  (0.279) | -0.110  (0.190) |
| Age 58+ | 0.352  (0.333) | -0.442  (0.455) | 0.001  (0.584) | -0.258  (0.306) | 0.344  (0.310) | -0.407  (0.823) | 0.076  (0.250) | -0.093  (0.197) |
| *Gender (Base: Male)* | | | | | | | | |
| Female | 0.107  (0.170) | 0.016  (0.230) | 0.092  (0.310) | 0.101  (0.166) | 0.304  (0.171) | 0.483  (0.254) | 0.079  (0.115) | 0.188  (0.101) |
| *Household Income (Base: below $30K)* | | | | | | | | |
| $30K- $50K | 0.048  (0.306) | -1.179^**^  (0.411) | -0.579  (0.357) | -0.177  (0.269) | 0.307  (0.298) | -0.346  (0.431) | -0.202  (0.188) | -0.047  (0.167) |
| $50K - $80K | -0.581^*^  (0.280) | -1.184^**^  (0.390) | -0.759  (0.475) | -0.200  (0.243) | 0.160  (0.295) | 0.064  (0.444) | -0.473^*^  (0.197) | -0.053  (0.159) |
| $80K - $100K | -0.417  (0.339) | -0.795^*^  (0.394) | -1.642^**^  (0.515) | -0.807^**^  (0.291) | 0.307  (0.316) | -0.737  (0.476) | -0.401  (0.211) | -0.210  (0.190) |
| $100K - $150K | -0.692^*^  (0.306) | -0.540  (0.515) | -2.475^**^  (0.766) | -0.140  (0.292) | 0.084  (0.341) | -0.696  (0.543) | -0.421  (0.244) | -0.025  (0.198) |
| $150K or more | -0.243  (0.328) | -0.735  (0.430) | -1.373^*^  (0.658) | -0.117  (0.263) | -0.297  (0.299) | -1.097^*^  (0.532) | -0.144  (0.216) | -0.088  (0.174) |
| *Ethnicity (Base: White)* | | | | | | | | |
| Asian | -0.101  (0.282) | 0.382  (0.352) | -0.478  (0.522) | -0.053  (0.262) | -0.274  (0.285) | 0.208  (0.509) | -0.118  (0.139) | 0.146  (0.134) |
| Other | 0.498  (0.278) | -0.355  (0.366) | -0.319  (0.519) | 0.244  (0.208) | 0.303  (0.326) | 0.535  (0.591) | 0.165  (0.182) | 0.135  (0.156) |
| Middle Eastern | -1.805^***^  (0.426) | 0.572^*^  (0.263) |  | -0.636  (0.428) | -0.508^**^  (0.193) |  | 0.533^*^  (0.236) | -0.259  (0.143) |
| *Estimation Diagnostics* | | | | | | | | |
| cut1 | -2.855^***^  (0.423) | -2.974^***^  (0.531) | -6.735^***^  (0.956) | -1.494^***^  (0.351) | -1.444^***^  (0.401) | -1.422  (0.741) | -2.807^***^  (0.326) | -1.275^***^  (0.227) |
| cut2 | -2.725^***^  (0.418) | -2.899^***^  (0.546) | -4.078^***^  (0.860) | -0.469  (0.337) | 0.059  (0.375) | 0.060  (0.729) | -2.256^***^  (0.287) | -0.159  (0.217) |
| cut3 | -1.965^***^  (0.399) | -2.833^***^  (0.549) | -3.028^***^  (0.770) | 0.312  (0.334) | 0.638  (0.373) | 0.942  (0.746) | -1.696^***^  (0.264) | 0.484^*^  (0.217) |
| cut4 | -0.632  (0.368) | -1.868^***^  (0.514) | -0.623  (0.714) | 1.485^***^  (0.349) | 2.410^***^  (0.402) | 2.680^***^  (0.799) | -0.534^*^  (0.240) | 1.838^***^  (0.235) |
| Observations | 207 | 181 | 94 | 207 | 181 | 94 | 482 | 482 |
| Pseudo R-Square | 0.211 | 0.222 | 0.505 | 0.054 | 0.134 | 0.328 | 0.268 | 0.092 |
| Chi-Square | 84.2*** | 48.4*** | 115.4*** | 30.1*** | 108.7*** | 132.2*** | 219.2*** | 108.6*** |
| p | 0.000 | 0.001 | 0.000 | 0.001 | 0.000 | 0.000 | 0.000 | 0.000 |

Standard errors in parentheses.* *P* < .05, ** *P* < .01, *** *P* < .001

Textbox A3. Details on an Ordered Probit Model

| An ordered Probit model is a method to conduct regression analysis designed for ordinal variables, where each ordered category represents different levels of response. The interval between levels may not necessarily be consistent, and standard regression analysis with continuous variables may not result in the correct estimates of parameters. The model assumes a set of thresholds exist, where each segment *i* between thresholds corresponds to the ordinal value of responses. A linear function of the independent variables, plus random error, estimates the probability of observing *i* within the range as follows:  *Pr(outcome_j_ = i) = Pr(κ_i_−1 < β_1_x_1j_ + β_2_x_2j_ + · · · + β_k_x_kj_ + u_j_ ≤ κ_i_)*  Where, *u*_j_ is assumed to be normally distributed, and cut points of the segment are denoted as κ_1_, κ_2_, . . . , κ_i−1_, where *i* is the number of response values. Using ordered Probit regression, we estimate to what extent our set of key variables influence SELF-ADHERENCE and OTHERS’-ADHERENCE separately. |
| --- |
